# Improvement of dysphagia in a child affected by Pompe disease treated with enzyme replacement therapy

**DOI:** 10.1186/1824-7288-39-30

**Published:** 2013-05-13

**Authors:** Simona Fecarotta, Serena Ascione, Giuseppe Montefusco, Roberto Della Casa, Paola Villari, Alfonso Romano, Ennio Del Giudice, Generoso Andria, Giancarlo Parenti

**Affiliations:** 1 Dipartimento di Scienze Mediche Traslazionali, Sezione di Pediatria, Università di Napoli Federico II, Naples, Italy; 2 Dipartimento di Otorinolaringoiatria e Scienze Affini, Università di Napoli Federico II, Naples, Italy

**Keywords:** Glycogen storage disease type II, Deglutition disorders, Enzyme replacement therapy

## Abstract

**Aim:**

Dysphagia is a known complication in Pompe Disease (PD), a severe metabolic myopathy due to alpha-glucosidase deficiency. Enzyme replacement therapy (ERT) with alglucosidase alfa is the only approved therapy for PD. Presently no data are available on the effects of ERT on dysphagia in PD patients. The aim of this work is to evaluate the course of this complication in a 6 years old boy affected by PD after treatment with ERT.

**Methods:**

Dysphagia was assessed by Videofluoroscopic Swallowing Study (VFSS) at baseline, before the start of ERT and after 36 months of therapy. We used the Dysphagia Severity Rating Scale (DSS) to define the severity grade of dysphagia.

**Results:**

VFSS performed at baseline revealed complete incoordination of oral stage swallowing which was classified as a grade 1 dysphagia according to DSS. After 36 months of treatment VFSS revealed normal swallowing, classified as grade 0 by DSS.

**Conclusion:**

Our results suggest that ERT is effective in improving dysphagia. VFSS may be a useful tool to investigate and monitor swallowing disorders in patients affected by PD.

## Background

Pompe Disease (PD) (OMIM 232300, glycogen storage disease type II, acid maltase deficiency) is a rare metabolic myopathy, due to the deficiency of the lysosomal acid-α-glucosidase (GAA) (E.C. 3.2.1.20) [1,2]. The overall incidence of PD is estimated 1 in 40,000 live births [3,4].

The deficiency of GAA leads to generalized glycogen accumulation, that is most relevant for the clinical manifestations in heart and skeletal muscles. The phenotype of the disease encompasses a continuum that ranges from severe, early-onset forms to attenuated, late-onset phenotypes. The severe end of this spectrum is traditionally referred to as the “classic infantile” form, which presents within the first months of life with severe and progressive hypotonia, hypertrophic cardiomyopathy, macroglossia, feeding difficulties, recurrent respiratory infections, and failure to thrive. If untreated, classic infantile patients generally die in the first year of life [5-7]. Non-classic infantile-onset forms share a similar clinical picture, but with milder or absent heart involvement, which may be limited to ECG abnormalities [8].

In the late-onset forms symptoms may present at any age from childhood to late adulthood, cardiac involvement is generally absent and the clinical manifestations are mostly related to skeletal muscle involvement, with progressive motor impairment, respiratory failure, and premature death.

Typical biochemical markers of PD are the elevated levels of transaminases and creatine kinase (CK) in blood, but these values vary considerably among different patients, and show broad fluctuations in time [9].

The definitive diagnosis of PD relies on the demonstration of reduced or absent GAA activity in lymphocytes, cultured fibroblasts, or muscle biopsies [10], and on the identification of mutations of the *GAA* gene.

At present, the only approved therapy for PD is Enzyme Replacement Therapy (ERT) with recombinant human alglucosidase alfa (Myozyme®, Genzyme Corporation, Cambridge, MA, USA). This therapy is based on periodic infusions of the recombinant enzyme, that is delivered to tissues where it replaces the defective GAA. Several clinical trials in classic infantile PD demonstrated remarkable success of this approach in correcting cardiac pathology and function and in extending patients’ survival, whereas the response of skeletal muscle was less striking [11-13]. ERT seemed to be more effective when started earlier [14].

Long-term administration of ERT has modified the natural course of PD, and different complications, previously unrecognized, have become evident [15], including conductive and neurosensorial hearing impairment [16], intracranial artery abnormalities [17], osteopenia and osteoporosis [18]. Recently new strategies for the treatment of PD are emerging [19].

Although feeding difficulties have been frequently reported and are commonly considered as part of the typical PD phenotype, only recently particular attention has been paid to the characterization of dysphagia and swallowing disorders [20-22]. These complications may substantially impact on the quality of life of patients and may lead to malnutrition, dehydration, weight loss and failure to thrive. In addition, airway invasion and aspiration may occur, increasing the risk of potentially lethal respiratory infections. Ineffective cough, due to the involvement of respiratory muscles, contributes to the risk of aspiration pneumonias.

Due to the severe consequences of dysphagia, its identification is relevant for the appropriate management of PD patients. Although at present there are no published studies on the effects of ERT on dysphagia, accurate and serial evaluations of swallowing may also be useful as markers to assess the efficacy of therapy in PD.

Dysphagia can be investigated with Videofluoroscopy Swallowing Study (VFSS) or with fibreoptic endoscopy. VFSS may be preferable as it is less invasive and easily tolerated, in infants and children. In addition, by VFSS it is possible to examine the bolus progression, to characterize the involvement of different stages of swallowing (oral, pharyngeal and esophageal), and to detect aspiration of contrast medium into the airways [23].

Here for the first time we present the data obtained in a patient with infantile-onset PD that was studied by VFSS before the start of ERT and after 36 months of therapy.

### Case report

CF, a 6-year-old boy, is affected by the nonclassic infantile form PD, and has received ERT with alglucosidase alfa (Myozyme^®^) since the age of 2.3 years.

He was born from healthy, non-consanguineous parents. Pregnancy was characterized by reduced fetal movements. Breast feeding difficulties and feeble cry were reported, since the first days of life. At 2 years of age he was referred to medical attention because of severe hypotonia, recurrent respiratory tract infections, macroglossia, open drooping, tent shaped mouth and failure to thrive. At that time he was able to sit, but was unable to stand.

Chewing and swallowing problems were also reported, that caused feeding difficulties and insufficient caloric intake.

Blood biochemistry showed increased serum levels of aspartate aminotransferase (approximately 7-fold the upper normal value), alanine aminotransferase (2.5-fold the upper normal range) and creatine kinase (3.7-fold the upper normal range). Electrocardiography showed short PR interval (80 msec).

A diagnosis of PD was suspected when he was 2.3 years old, and confirmed by GAA enzymatic assay in lymphocytes and fibroblasts. The molecular analysis of the *GAA* gene showed the mutations [c.-32-13 T > G] and [c.1856 G > A] on one allele and [c.1655 T > C] on the other allele.

Due to the reported chewing and swallowing difficulties, a VFSS was performed before the start of ERT, by administering oral boluses of 5 ml liquid barium according to standard (published) procedures [23]. VFSS showed complete incoordination of the oral stage swallowing with anterior positioning of bolus, weakness of labial and oral cavity muscles and delayed bolus transit. Weak and incoordinated tongue motion was ineffective for swallowing with spilling of barium boluses from the mouth. Epiglottis deflection was normal and pharyngeal and esophageal stages of swallowing were regular. No airway invasion was evident.

According to a dysphagia severity scale (DSS) (adapted by Gates et al. [24]), dysphagia was limited to oral stage and classified as grade 1 severity (Table 1). A supportive speech therapy was started.

**Table 1:** **Dysphagia Severity Rating Scale (adapted by Gates et al.**[23]**)**

**0**	**Normal swallowing**	
1	Minimal dysphagia	VFFS shows a slight deviation from a normal swallow; impairment limited to oral stage
2	Mild dysphagia swallowing suggestion	Presence of oro-pharyngeal dysphagia; can be treated by means of specific swallowing suggestion
3	Mild to moderate dysphagia	Presence of oro-pharyngeal dysphagia potential for aspiration exists but can be diminished by specific swallowing techniques
4	Moderate dysphagia	Presence of oro-pharyngeal dysphagia; potential for aspiration exists: trace aspiration can be seen at VFSS
5	Severe dysphagia	Presence of aspiration; nothing by mouth is recommended

ERT with alglucosidase alfa was started at the age of 2.3 years at a dose of 20 mg/kg/14 days. The patient was periodically monitored.

At the age of 6.3 years, after 36 months of ERT, no progression of clinical manifestations related to muscle involvement was observed and the general clinical picture seemed stable. Physical examination still showed generalized hypotonia and weak tendon reflexes. The increase of serum transaminases and creatine phosphokinase persisted. Recurrent pneumonias were reported, but the patient never required invasive ventilation. Facial-muscle weakness with open drooping mouth, expressionless face, absence of horizontal forehead lines, ptosis of the eyelid persisted. Hearing loss, impaired speech with articulatory disorders and hypernasal resonance were also evident. The feeding difficulties were improved and no additional problems in drinking or eating food of any consistence were reported.

Swallowing ability was again assessed by VFSS at the age of 6.3 years. Substantial improvement of swallowing mechanisms was observed, that correlated with the reported absence of feeding difficulties. Barium boluses were regularly inserted without spillage. The oral stage was regular and oral acceptance was coordinated. Tongue thrusts were coordinated and effective to push the food. Epiglottis deflection was regular. The transit of barium contrast medium was normal pharyngeal and esophageal phases. In summary, VFSS revealed the absence of dysphagia, that classified as grade 0 by the DSS score (Table 1).

## Discussion

This is the first case of a single PD infantile patient with a documented improvement of dysphagia after three years of ERT. The improvement of dysphagia was assessed by VFSS, performed before the start of ERT and after 36 months of treatment, and by a DSS scoring scale. Dysphagia has already been reported in PD patients. A study of 13 untreated children with classic infantile PD, showed that VFSS abnormalities were detectable in all patients, including a 15-day-old infant [20]. In this study the severity of dysphagia was rated on the basis of clinical information, and of the need for special dietary recommendations (normal foods, modified foods, or non-oral nutrition). In this group of patients a second assessment of VFSS was not performed after the start of ERT.

Van Gelder’s et al. [21] examined the course of dysphagia in four patients on ERT, by fibreoptic endoscopy. By this approach, dysphagia apparently remained stable during treatment in three patients and worsened in one. ERT seemed to reduce feeding difficulties in some patients, who could discontinue nasogastric tube feeding, but there was no complete normalization of swallowing, even in patients who were orally fed. In this study no data before the start of ERT were available.

In our patient, at diagnosis, VFSS showed a weak and incoordinated swallowing during the oral phase, a finding already reported in PD patients [20]. After 36 months of therapy VFSS showed coordinated swallowing mechanisms, with effective deglutition. The DSS improved from 1 to 0. Although this was a subtle change, it may be clinically relevant in a progressive disease like PD in which symptoms worsen over time without treatment.

These results suggest that ERT (possibly in combination with supportive speech therapy) may be useful to improve swallowing in PD patients, although this observation in a single child should be confirmed in larger numbers of patients and in formal clinical studies.

It is known that PD manifestations have a broad severity spectrum and respond variably to therapy. The identification of reliable measures to follow disease progression and ERT efficacy has been so far a challenging task. Biochemical markers of disease severity are unreliable, and administration of functional tests can be difficult in young patient, or may be influenced by the operator. VFSS is a safe and non invasive method to investigate dysphagia, which can be performed in children by skilled radiologists [23,24]. VFSS is also useful to get a quantitative severity score of dysphagia with appropriate scales, and to identify airways invasion or risk for aspiration. If further studies in larger populations will confirm our data, one could hypothesize that an accurate evaluation of dysphagia may represent an additional marker to monitor ERT efficacy.

The pathophysiology of dysphagia in PD patients is not completely defined. It is possible to speculate that different mechanisms may concur in causing abnormal swallowing. These include the dysfunction of bulbar muscles and the weakness of respiratory muscles and diaphragm that can lead to impaired coordination of swallowing and breathing. In addition, recent studies pointed to a possible role of the involvement of lower motor neurons [20].

In principle, all these mechanisms may coexist in our patient. However, an effect on the motor neurons can be excluded, as it is known that ERT cannot cross the blood–brain-barrier and reach therapeutic levels in the central nervous system. Therefore, it would be reasonable to think that the improvement of dysphagia during ERT reflects an improvement of bulbar muscle and diaphragm function.

## Conclusions

In conclusion, we suggest that VFSS and the VFSS-based score scale are useful tools in the management of PD patients, to document the presence of dysphagia and to reduce the risk of life-threatening complications. In our patient only two observations were available, before the start of ERT and 36 months later. It would be desirable for the management of PD patients to include serial, periodical VFSS evaluations, in order to monitor the evolution of dysphagia during treatment and to start early appropriate supportive measures. Speech therapy, may be considered, as an adjunctive therapy, in a multidisciplinary approach for the treatment of PD patients [25]. Further studies in larger groups of patients are needed to confirm the possible role of ERT to improve dysphagia.

### Consent

Written informed consent was obtained from the patient for publication of this Case report and any accompanying images. A copy of the written consent is available for review by the Editor-in-Chief of this journal.

## Abbreviations

PD: Pompe disease; ERT: Enzyme replacement therapy; VFSS: Videofluoroscopic swallowing study; DSS: Dysphagia severity rating scale; GAA: Acid-***α***-glucosidase; CK: Creatine kinase

## Competing interests

Giancarlo Parenti has received travel support from Genzyme, Generoso Andria has received unrestricted research grants, honoraria and travel support from Amicus, Genzyme, Shire, Biomarin and Actelion. Simona Fecarotta has received travel support from Genzyme and Actelion.

## Authors’ contribution

SF has made substantial contributions to conception and design, analysis and interpretation of data, has been involved in drafting the manuscript and has given final approval of the version to be published. SA has made substantial contributions in the acquisition of data, has been involved in drafting the manuscript, and has given final approval of the version to be published. GM has made substantial contributions to acquisition of data has been involved in drafting the manuscript, and has given final approval of the version to be published. RDC has made substantial contributions to acquisition of data, has been involved in revising the manuscript critically for important intellectual content, and has given final approval of the version to be published. PV has made substantial contributions to conception and design, has been involved in revising the manuscript critically for important intellectual content, and has given final approval of the version to be published. AR has made substantial contributions to analysis and interpretation of data, has been involved in revising the manuscript critically for important intellectual content, and has given final approval of the version to be published. EDG has made substantial contributions to analysis and interpretation of data, has been involved in revising the manuscript critically for important intellectual content, and has given final approval of the version to be published. GA has made substantial contributions to conception and design and analysis and interpretation of data, has been involved in revising the manuscript critically for important intellectual content, and has given final approval of the version to be published. GP has made substantial contributions to conception and design and analysis and interpretation of data, has been involved in drafting the manuscript and revising it critically for important intellectual content, and has given final approval of the version to be published. All authors read and approved the final manuscript.
